# Dual Disease Burden: Growing Older with Congenital Heart Disease and Hereditary Metabolic and Connective Tissue Disorders—Data from the PATHFINDER-CHD Registry on Heart Failure

**DOI:** 10.3390/geriatrics10060152

**Published:** 2025-11-20

**Authors:** Ann-Sophie Kaemmerer-Suleiman, Frank Harig, Annika Freiberger, Oliver Dewald, Stephan Achenbach, Aysenur Akyol, Helena Dreher, Anna Engel, Peter Ewert, Sebastian Freilinger, Jürgen Hörer, Christopher Hohmann, Stefan Holdenrieder, Robert David Pittrow, Harald Kaemmerer, Renate Kaulitz, Frank Klawonn, Christian Meierhofer, Steffen Montenbruck, Nicole Nagdyman, Rhoia Neidenbach, Elsa Ury, Leonard Bernhard Pittrow, Benjamin Alexander Pittrow, Fabian von Scheidt, Nicole Wolfrum, Michael Huntgeburth, Pelagija Zlatic, Mathieu N. Suleiman, Fritz Mellert

**Affiliations:** 1 Department of Cardiac Surgery, University Hospital Erlangen, Friedrich-Alexander-University Erlangen-Nürnberg, Krankenhausstrasse 12, 91054 Erlangen, Germanyaysenur.akyol@fau.de (A.A.);; 2 International Center for Adults with Congenital Heart Disease, Department of Congenital Heart Disease and Paediatric Cardiology, German Heart Center Munich, University Hospital of the Technical University, 80636 Munich, Germanyewert@dhm.mhn.de (P.E.); fabian.scheidt@tum.de (F.v.S.); wolfrumn@dhm.mhn.de (N.W.);; 3 Department of Cardiology, Friedrich-Alexander-University Erlangen-Nürnberg, 91054 Erlangen, Germany; 4 Chair of Preventive Pediatrics, Department Health and Sport Sciences, School of Medicine and Health, Technical University Munich, 80333 Munich, Germany; 5 Department of Congenital and Paediatric Heart Surgery, German Heart Center Munich, University Hospital of the Technical University, 80636 Munich, Germany; 6 Division of Congenital and Pediatric Heart Surgery, European Kids Heart Center, University Hospital Großhadern, Ludwig-Maximilians-University Munich (LMU), 81377 Munich, Germany; 7 Clinic III for Internal Medicine, Faculty of Medicine, University Hospital Cologne, University of Cologne, 50937 Cologne, Germany; christopher.hohmann@uk-koeln.de; 8 Department of Laboratory Medicine, German Heart Center Munich, University Hospital of the Technical University, 80636 Munich, Germany; 9 Pediatric Cardiology, Universitätsklinikum Tübingen, 72076 Tübingen, Germany; 10 Helmholtz Centre for Infection Research, Biostatistics, Technical University Braunschweig, 38124 Braunschweig, Germany; 11 Department of Sport and Human Movement Science, University of Vienna, 1010 Vienna, Austria

**Keywords:** adult congenital heart disease, heart failure, aging, geriatrics, registry, PATHFINDER-registry

## Abstract

**Background:** Advances in diagnosis and treatment have led to a growing population of adults with congenital heart disease (ACHD). Despite increasing life expectancy, their clinical needs—especially in older age—remain poorly defined. Cardiac and non-cardiac comorbidities are prevalent, and emerging evidence suggests accelerated biological aging compared to the general population. However, data on older patients and geriatric patients with CHD are limited. **Objectives:** This study aimed to characterize patients with CHD aged ≥50 years, focusing on functional status, comorbidities, sex-specific differences, and therapeutic patterns. **Methods:** The PATHFINDER-CHD Registry is a prospective, observational, multicenter registry enrolling patients with CHD with manifest heart failure (HF), HF history, or high HF risk. Data include anatomy, prior treatments, comorbidities, and medication use. **Results:** Among 1935 patients, 297 were ≥50 years old. Most had acyanotic CHD (62%); Tetralogy of Fallot (21%) was the most frequent diagnosis. A morphologic right systemic ventricle was present in 12%, and 5% had univentricular hearts. HF was manifest in 21%; 44% were classified as ACC/AHA stage B, 51% as stage C, yet 77% were in Perloff class I/II. Common cardiovascular comorbidities included aortopathy (55%), hypertension (37%), and arrhythmia (33%). Non-cardiac comorbidities included thyroid dysfunction (25%), renal impairment (18%), and neurological disease (13%). Sex-specific differences were observed. Despite HF burden, SGLT2 inhibitors and ARNIs were used in only 17% and 8.4%, respectively. **Conclusions:** Older patients with CHD represent a clinically complex cohort with high comorbidity burden. The findings support the concept of accelerated aging and emphasize the need for tailored interdisciplinary care strategies.

## 1. Introduction

Congenital heart disease (CHD) is the most common congenital anomaly, affecting approximately 1 in 100 live births [1]. Over the past few decades, significant advances in cardiology, pediatric cardiology, congenital heart surgery, and perioperative intensive care have resulted in a dramatic improvement in life expectancy for individuals with CHD. Today, more than 95% of children born with CHD survive in the industrialized world into adulthood. Meanwhile, the number of adults with congenital heart disease (ACHD) even exceeds that of pediatric patients [2]. As this population continues to age, a growing number of individuals are reaching not only middle age but also older and geriatric stages of life [3,4,5].

This demographic shift brings forth new clinical challenges. Patients with CHD are no longer defined solely by their congenital anatomy but increasingly by a complex interaction between residua from the CHD, prior surgical or interventional treatment, and acquired cardiovascular comorbidities. Many have undergone multiple cardiac surgeries during childhood and early adulthood. Over time, bioprosthetic valves, conduits, and surgical reconstructions may deteriorate, requiring reoperations or percutaneous reinterventions and the need for dedicated care and treatment with repeat interventions becomes more frequent.

Furthermore, with age, cardiac and non-cardiac comorbidities such as cardiac arrhythmias, pulmonary hypertension, aortopathies, systemic arterial hypertension, coronary artery disease, and chronic organ diseases, such as cerebrovascular, hepatic, renal, vascular, hematological and endocrinologic diseases become increasingly prevalent, further complicating clinical management.

Moreover, advancing age is associated with an increasing prevalence of both cardiac and non-cardiac comorbidities, including cardiac arrhythmias, pulmonary hypertension, aortopathies, systemic hypertension, coronary artery disease, and chronic organ dysfunction. These affect the cerebrovascular, hepatic, renal, vascular, hematologic, and endocrine systems, and complicate clinical management further. In this context, heart failure (HF) has emerged as one of the leading causes of morbidity and mortality. Longstanding hemodynamic burdens from surgical residua and sequelae, ventricular dysfunction or arrhythmias predispose ACHD to HF [6,7]. This burden continues to rise with advancing age due to the additive effects of acquired disease.

Despite the growing number and complexity of older patients with CHD, they remain underrepresented in large HF trials and contemporary cardiovascular guidelines. Conventional HF management strategies developed for patients with acquired heart disease often do not reflect the specifics of congenital anatomy, surgical history, or lifelong disease burden. Likewise, ACHD guidelines offer little guidance on integrating age-related care concepts such as multimorbidity management, frailty assessment, and rehabilitation planning in the context of aging [8,9,10].

To address this evidence gap, patients with CHD were consecutively enrolled in the PATHFINDER-CHD registry, a prospective registry [11,12]. In this study, we provide a focused analysis of patients with CHD aged ≥50 years enrolled in the registry. Our aim is to characterize the clinical profile of this under-recognized population, describe their comorbidities and HF phenotypes, and highlight specific challenges such as re-interventions, and the need for age-adapted care models.

## 2. Materials and Methods

In the present study, the focus was placed on aging and geriatric patients with CHD (defined as those aged 50 years and older) to determine how age impacts their clinical profile, comorbidities, and HF burden. Patients were enrolled between 2 November 2022, and 20 May 2025.

The PATHFINDER-CHD Registry is a prospective, observational, web-based HF-registry established in Germany in 2022 to capture and characterize adults with CHD or hereditary connective tissue disease and HF.

This multicenter initiative is a collaboration between the International Center for Adult Congenital Heart Disease at the German Heart Center Munich, the Departments of Cardiac Surgery and Cardiology at Friedrich-Alexander-University Erlangen-Nürnberg, the Department of Pediatric Cardiology at University Hospital Tübingen, and the Department of Cardiology at the University of Cologne.

Included were ACHD with manifest HF (clinically or diagnostically confirmed), a documented history of HF, or significant structural or functional risk factors for developing HF.

Patients were enrolled consecutively and without preselection at the participating institutions during their regular check-ups or hospital stays.

Based on the recommendations of the American College of Cardiology (ACC) and the American Heart Association (AHA), an adapted heart failure classification tailored to the specific characteristics of congenital heart disease (CHD), which differ from those of other medical conditions, was applied [13]. Accordingly, HF was divided into four stages. Stage A includes individuals at high risk of developing HF but without structural heart disease or symptoms. Stage B includes those with structural heart disease but without symptoms. Stage C includes those with structural heart disease and current or past symptoms. Stage D includes those with advanced HF and persistent symptoms despite medical therapy.

The functional class (FC) of patients with CHD was determined according to Perloff’s classification, which classifies patients with CHD based on their symptomatic restrictions [3].

Demographic and clinical data were obtained through a systematic review of medical records and documented using a structured case report form. Recorded variables included age, sex, underlying CHD diagnosis, heart failure classification, comorbidities, anthropometric measurements (height, weight, BMI), oxygen saturation, medication use, and prior surgical or interventional cardiac treatments.

### 2.1. Ethics Approval and Consent

The study was approved by the institutional ethics committees of the Technical University of Munich, the Friedrich-Alexander-University Erlangen-Nürnberg, the university of Cologne, the university of Tübingen. Written informed consent was obtained from all participants. The study complies with good pharmacoepidemiologic practice (GPP) and current data protection regulations.

### 2.2. Statistical Analysis

Statistical analyses were performed using R version 4.4.3. Data were pseudonymized for analysis. Numeric variables were described using the mean ± standard deviation, median, and range (minimum and maximum). Numerical parameters were compared by Wilcoxon rank sum test, categorical parameters by Pearson’s Chi-squared test and Fisher’s exact test. Statistical significance was determined as *p* < 0.05.

## 3. Results

### 3.1. Study Cohort

A total of 1935 patients with CHD patients were included in the study, of whom 297 were aged 50 years or older. The cohort comprised 163 women (54.9%) and 134 men (45.1%), without a statistically significant difference in sex distribution at the 5% significance level.

### 3.2. Demographics

The mean age was 57.9 ± 6.9 years (median 55.8; range: 50.1–82.8), with female participants being slightly older than male. The mean height was 170.5 ± 10.1 cm (median 170.0; range: 134.0–197.0), and the mean weight was 76.1 ± 16.5 kg (median 75.0; range: 37.0–166.0), resulting in a mean body mass index (BMI) of 26.1 ± 4.6 kg/m^2^ (median 25.4; range: 17.3–54.2). Male participants were significantly taller and had a higher BMI compared to female participants. Demographic characteristics of the study population are summarized in Table 1.

### 3.3. Age Distribution

**Figure 1:**
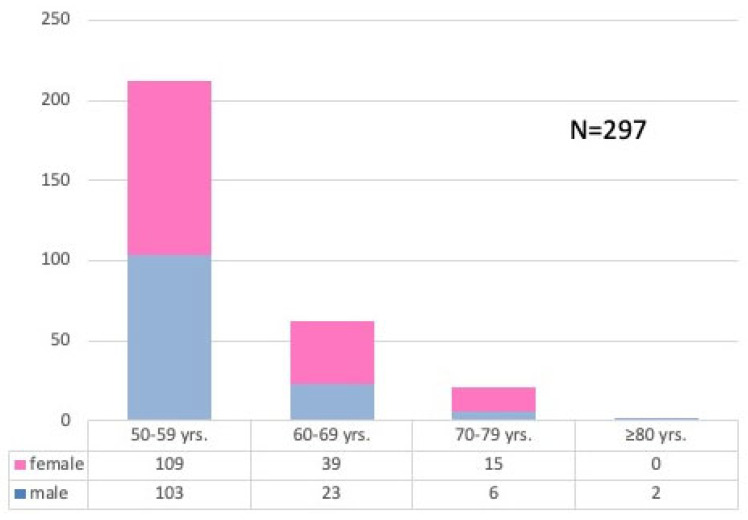
Distribution of patients by age group and sex.

Most of the 297 patients were in their 6th decade (age group 50–59 years: n = 212; 109 female, 103 male) and 7th decade (age group 60–69 years: n = 62; 39 female, 23 male), while only a few were in the 8th decade (age group 70–79 years: n = 21; 15 female, 6 male) or beyond (age group ≥ 80 years: n = 2; both male) (Figure 1).

### 3.4. Underlying Congenital Heart Defect

The ACHD population consisted of a wide range of repaired or native CHD, including conotruncal defects (such as tetralogy of Fallot, pulmonary atresia-VSD), shunt lesions (such as atrial or ventricular septal defects), right heart anomalies (such as Ebstein’s anomaly or pulmonary valve or pulmonary artery disease), left heart anomalies (aortic valve disease, aortic coarctation), connective tissue or systemic disease and complex CHD (such as complete or corrected transposition, univentricular hearts).

The most common underlying congenital diagnoses were Tetralogy of Fallot (n = 62; 21%), Ebstein’s anomaly (n = 36; 12%), coarctation of the aorta (n = 32; 11%), valvular aortic stenosis (n = 22; 7.0%), and transposition of the great arteries (n = 20; 6.7%) (Table 2).

Most patients had a morphologic left systemic ventricle (n = 245; 82%), while 37 (12%) had a morphologic right systemic ventricle and 14 (4.7%) a univentricular heart (one was undetermined).

The majority of patients had a primarily acyanotic congenital heart defect (n = 183; 62%), while 114 patients (38%) had a primarily cyanotic congenital heart defect. Among those with initially acyanotic lesions, 8 patients (2.7%) developed secondary cyanosis over time due to Eisenmenger reaction (Table 2).

### 3.5. Prior Treatment

Regarding prior treatment, only 51 (17%) were treatment naïve. A total of 219 patients (74%) had undergone reparative cardiac surgical repair, 9 (3.0%) palliative operative treatment (e.g., pulmonary artery banding or aorto-pulmonary shunts), and 18 (6.1%) had received primary interventional therapy. There was no significant difference between men and women (*p* = 0.7) (Table 3).

### 3.6. Clinical Data

A total of 61 (21%) patients had manifest HF, while 234 (79%) were considered at risk for HF.

**Figure 2:**
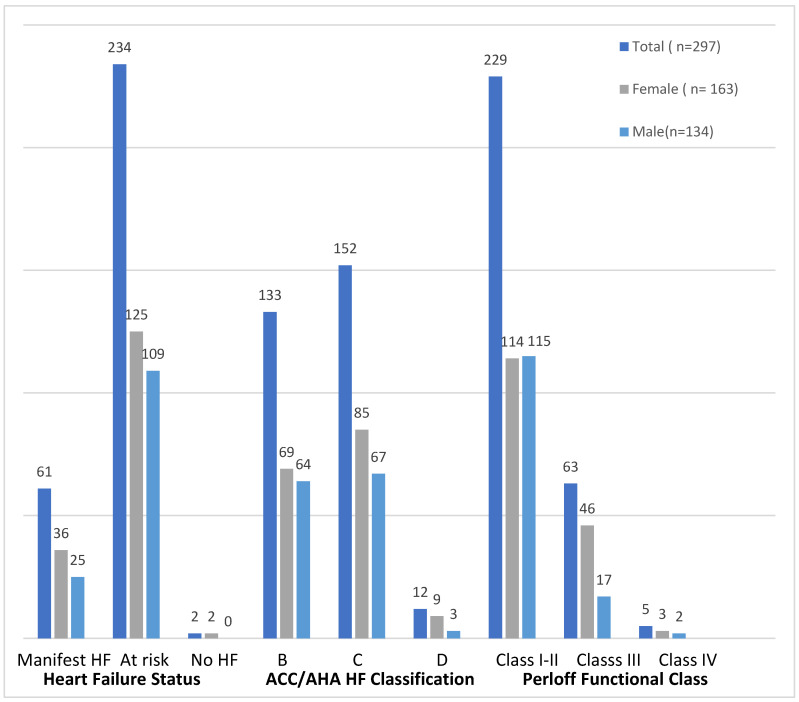
Distribution of heart failure status, ACC/AHA classification, and Perloff functional class in the study population (total, female, and male).

According to the modified ACC/AHA-HF classification, most patients were classified as C (n = 152; 51%) or B (n = 131; 44%), with 12 (4%) classified as D. In terms of functional capacity, 229 (77%) patients were in Perloff Functional Class I/II, 63 (21%) in class III, and only five (1.7%) in class IV (Figure 2).

### 3.7. Cardiac and Non-Cardiac Comorbidities

Cardiac and non-cardiac comorbidities were common. Cardiac comorbidities in men included particularly aortopathies (65%) or systemic arterial hypertension (41%). In women arrhythmias (38%) and pulmonary hypertension (8%) were more prevalent. 3% had a history of previous infective endocarditis.

Non-cardiac comorbidities included in the entire cohort particularly thyroid dysfunction (25%), hyperlipidemia (19%), renal failure (18%), neurological disorders (13%), hyperuricemia (13%), anemia (10%) and liver dysfunction (10%) (Table 4).

### 3.8. Pharmacotherapy

Many patients (90%) received pharmacological treatment. The most commonly prescribed drugs for cardiac reasons were beta-blockers (62%), AT1-antagonists (35%), diuretics (35%), and mineralocorticoid receptor antagonists (MRAs) (25%) (Table 5).

For the treatment of HF, modern SGLT2 inhibitors or angiotensin receptor-neprilysin inhibitors (ARNI) were used in 17% and 8.4%, respectively. Only 6.1% of patients were still receiving digitalis glycosides. Anticoagulation was used in 52% of patients. More than 31% of patients were on thyroid hormone substitution.

## 4. Discussion

The number of patients with CHD has steadily increased over recent decades and now surpasses the number of affected children. Primarily due to advances in congenital cardiology and congenital heart surgery, even patients with complex CHD are frequently reaching middle and older age [14]. This demographic shift presents new challenges for healthcare delivery [3].

Management of patients with CHD in later life is complicated by the wide variety of underlying defects, diverse histories of surgical or interventional treatment, and the high prevalence of late complications. At the same time, accumulating evidence indicates that this patient population is affected earlier in life by health issues that, in the general population, occur at a higher biological age; patients with CHD show an earlier manifestation of age-related comorbidities, including HF, arrhythmias, liver and kidney disease, neurological complications, and endocrine disorders [5].

While most previous studies have focused on younger adults, systematic data on older patients with CHD with manifest HF or the risk of developing HF remains limited. The present study analyzes a cohort of 297 adult patients with CHD or hereditary connective tissue disease aged 50 and older from the Pathfinder-CHD registry, aiming to describe structural, functional, and clinical heterogeneity of this population [11,12]. Particular attention is given to cardiac function and comorbidity burden to derive implications for diagnosis, therapy, and long-term care. This represents one of the largest systematic characterizations of an aging ACHD population with HF to date. Our data highlight the increasing demographic relevance of this group and the complexity of their healthcare needs, confirming key aspects described in current literature on geriatric care of patients with CHD [15].

This study provides one of the largest descriptive analyses of adults with congenital heart disease aged 50 years and older. These patients represent a growing yet underrecognized subgroup that exists because of decades of advances in congenital cardiac surgery and cardiology. Despite these advances, more than half of the cohort were classified as ACC/AHA stage C—indicating current or prior heart-failure symptoms—and multimorbidity and polypharmacy were frequent. Our objective was not causal inference but to offer a real-world clinical profile of older patients with CHD currently in care and to establish a reference point for clinicians and investigators.

Because this is a cross-sectional snapshot without a comparison group, and because treatment strategies have evolved across eras, our findings should be interpreted as a contemporary benchmark rather than a statement of secular trends or uniqueness versus other populations. The key message is that older patients with CHD carry the dual burden of residua or sequelae of their congenital lesions and age- or lifestyle-related comorbidities. This combination heightens risks of functional decline, heart-failure progression, and healthcare utilization, underscoring the need for tailored, multidisciplinary care models that integrate adult congenital cardiology, cardiac surgery, and geriatrics. Future work should build on these descriptive data using longitudinal designs and explicit comparators, such as younger patients with CHD and age-matched general-population cohorts. Moreover, it should evaluate potential differences in the management and response to heart-failure therapies in elderly patients with CHD.

In the following, we detail patterns of biological aging, lesion complexity, sex-specific differences, comorbidities, and therapeutic use observed in this cohort.

### 4.1. Aging and Morbidity Burden: Biological Versus Chronological Age

As emphasized by Bonanni et al. (2025) and Tournoy et al. (2023), aging in ACHD is marked by a discrepancy between chronological and biological age [16,17]. In our cohort, age-related comorbidities were already prevalent at a mean age of just 57.9 years, reflecting the concept of “accelerated aging” driven by lifelong hemodynamic stress, surgical trauma, and chronic inflammation [17]. More than one-fifth of patients had manifest HF, and another 70 were classified as “at risk”, a high rate compared to the general population at this age.

### 4.2. Clinical Heterogeneity and Structural Complexity

Our cohort included a broad spectrum of CHD, with Tetralogy of Fallot, Ebstein’s anomaly, aortic coarctation being particularly common. The spectrum is expanded by patients with connective tissue disease, such as Marfan syndrome. The high proportion of Ebstein patients is attributable to participation by a specialized referral center for Ebstein’s anomaly. Notably, nearly 10% of patients had complex lesions such as ccTGA or univentricular hearts. These diagnoses were rarely compatible with survival beyond adolescence until recently. The high rate of cardiac surgical pre-treatment (74%) and a prevalence rate of 38% of patients with primary cyanotic lesions illustrate the significant structural burden but also the success of earlier therapeutic interventions. Nevertheless, our findings support previous observations that many patients with CHD are treated but not cured and face increasing complications with age [18].

### 4.3. Sex-Specific Differences

Our data reveal significant sex differences in anthropometry, comorbidity profiles, and functional status. Men were more frequently affected by aortopathies and arterial hypertension, whereas women had higher rates of arrhythmias, pulmonary hypertension, and thyroid dysfunction. The latter, affecting 31% of women, may be due to hormonal, autoimmune, or iatrogenic factors, and remains underrepresented in ACHD literature. The high rate of aortopathies in men may also be influenced by hormonal factors, as it is well known from data on aortic aneurysm in the normal population.

### 4.4. Comorbidities and Medication Use

Cardiac and non-cardiac comorbidities were common, particularly thyroid dysfunction (25%), renal impairment (18%), neurological disease (13%), anemia, and liver dysfunction (10% each). These distributions align with the findings of Tournoy et al., who describe systemic premature aging in patients with CHD [17]. The medication profiles also reflect complex management needs. A total of 62% received beta-blockers, 35% AT1 antagonists, 60% diuretics and/or MRAs, and 52% anticoagulation. Modern heart failure therapies such as SGLT2 inhibitors (17%) and ARNIs (8.4%) were underutilized, possibly due to limited evidence in ACHD. While evidence remains limited, our data proves consideration of therapies like sacubitril/valsartan and SGLT2 inhibitors even for ACHD with systemic ventricular dysfunction. Sacubitril/valsartan, for example, appears tolerable in small ACHD cohorts [19,20,21,22,23]. Similarly, SGLT2 inhibitors have promising effects, particularly in Fontan circulation [24], but routine use awaits further validation [25]. The frequent use of thyroid hormone therapy (31%) further underscores the relevance of endocrine comorbidities.

### 4.5. Discrepancy Between Heart Failure Severity and Perceived Functional Status

A notable observation is the discordance between objective HF severity (mod. ACC/AHA classification) and perceived functional capacity according to Perloff. While 55% of patients were classified in ACC/AHA stage C or D, 77% reported Perloff functional class I or II status. This illustrates that even patients with complex CHD can maintain a favorable quality of life when managed through dedicated specialized care. However, anatomical complexity alone is not determinative of outcomes. Rather, the timing, quality, and continuity of care play a pivotal role [26]. This fact is often overlooked, as many studies emphasize lesion complexity over care quality. As a result, patients with ostensibly “simple” lesions may experience significant late complications when diagnosed or treated late, while those with complex pathoanatomy may achieve good outcomes if managed timely and effectively in a multidisciplinary team approach, particularly by world class congenital cardiac surgeons.

### 4.6. Integration of Broader Clinical Evidence

Our findings are consistent with broader data, showing that patients with CHD are now surviving into their sixth, seventh, or even ninth decades [3,4]. This introduces new challenges not only for cardiologists and cardiac surgeons but also for primary care providers and specialists for prevention and rehabilitation [6,7]. These patients often present with a dual disease burden, consisting of both congenital sequelae (e.g., systemic right ventricles, residual shunts, cyanosis, aortopathies) and typical age-related comorbidities [27]. This unique heart failure phenotype is rarely addressed in guidelines based on acquired cardiac disorders [8,9,10].

Importantly, the need for structured lifelong follow-up in certified ACHD centers is reinforced by our data [28]. These centers provide critical oversight in detecting progressive dysfunction, planning interventions, managing transitions into advanced HF therapies, and offering comprehensive care that includes physical, cognitive, and psychosocial rehabilitation [29]. Without such oversight, patients are at risk of being lost to follow-up or excluded from appropriate services.

In addition, the growing complexity of the aging ACHD population must be recognized by healthcare systems and policymakers. These patients often live with residua from prior cyanosis, surgery or arrhythmias, and require highly individualized, multidisciplinary care that combines adult congenital cardiology, heart failure expertise, geriatric principles, and rehabilitation. Unfortunately, today only a few specialized centers offer such integrated care.

Preventive, rehabilitative, and health-promoting measures should be integrated into long-term care at an early stage and to a greater extent in order to improve the quality of life and prognosis of those affected.

In addition, future studies should specifically address potential differences in the management and response to chronic heart failure therapies in elderly CHD patients, as their lifelong exposure to congenital and acquired cardiovascular alterations may modify treatment efficacy and tolerance compared to the general population.

It is important to note that cardiac and/or non-cardiac comorbidities in patients with CHD often cannot be treated in the same way as acquired heart diseases. Examples of this include the management of patients with a morphologically right systemic ventricle or patients with univentricular hearts, for example, after Fontan surgery.

### 4.7. Limitations

This study is subject to several limitations. First, its cross-sectional design does not allow for conclusions regarding disease progression, causality, or long-term outcomes. Future longitudinal data from ongoing registries will be required to confirm these observations. Second, this cohort was derived from few tertiary care centers with high ACHD expertise, which may limit generalizability to broader populations or less specialized settings.

Third, although our analysis emphasizes biological aging and functional vulnerability, it did not include standardized biomarkers of biological age (e.g., epigenetic clocks, telomere length, inflammatory markers), which are needed to objectively quantify the mismatch between chronological and physiological aging in patients with CHD.

Finally, data on frailty, neurocognitive status, and quality of life were not systematically collected and should be integrated in future prospective studies.

## 5. Conclusions

Older patients with CHD represent a distinct and increasingly relevant subgroup in cardiovascular medicine. As survival into older age becomes the norm, the clinical focus shifts from surgical success to the long-term management of a complex interplay between residual congenital lesions, acquired comorbidities, and accelerated biological aging. Our findings confirm that even patients with complex CHD can maintain good functional capacity, particularly when care is continuous, interdisciplinary and personalized.

However, traditional paradigms of disease severity based solely on anatomical complexity fall short in capturing the full clinical picture. Delayed or inadequate treatment of seemingly simple defects may lead to significant late complications, while early, specialized care can substantially improve the long-term outlook even for patients with complex malformations.

Tailored follow-up strategies in experienced ACHD centers, improved integration of modern HF therapies, and a shift toward personalized, complexity-based care pathways are essential to address the needs of this rapidly expanding and vulnerable population. A better understanding of biological aging mechanisms and the inclusion of functional and psychosocial outcomes in future research should further improve care and prognosis.

## Figures and Tables

**Table 1:** Demographics of study population.

	Total (n = 297)	Female (n = 163)	Male (n = 134)	*p*-Value
**Age [years]**	57.9 ± 6.9 (55.8, range: 50.1–82.8)	58.5 ± 7.1 (57.3, range: 50.1–79.1)	57.1 ± 6.4 (55.1, range: 50.1–82.8)	0.081
**Height [cm]**	170.4 ± 10.3 (170.0, range: 134.0–197.0)	164.6 ± 8.2 (165.0, range: 134.0–183.0)	177.6 ± 7.7 (178.0, range: 153.0–197.0)	<0.001 *
**Weight [kg]**	76.1 ± 16.5 (75.0, range: 37.0–166.0)	69.1 ± 13.5 (68.0, range: 37.0–110.0)	84.6 ± 15.8 (82.0, range: 56.0–166.0)	<0.001 *
**BMI [kg/m^2^]**	26.1 ± 4.6 (25.4, range: 17.3–54.2)	25.5 ± 4.5 (24.8, range: 17.4–41.6)	26.8 ± 4.8 (25.9, range: 17.3–54.2)	0.011 *
**Oxygen saturation at rest [%]**	94.9 ± 5.5 (96.0, range: 54.0–99.0)	94.1 ± 6.8 (96.0, range: 54.0–99.0)	95.8 ± 2.8 (96.0, range: 83.0–99.0)	0.3
**Unknown**	20	8	12	

* statistically significant (*p* < 0.05).

**Table 2:** Leading Congenital Heart Defect, systemic disorders or hereditary connective tissue disease.

Congenital Cardiac Anomaly	Overall n = 297 ^1^	Female n = 163 ^1^	Male n = 134 ^1^
Aortic aneurysm, congenital	1 (0.3%)	0 (0%)	1 (0.7%)
Aortic stenosis, subvalvular	4 (1.3%)	2 (1.2%)	2 (1.5%)
Aortic stenosis, supravalvular	1 (0.3%)	0 (0%)	1 (0.7%)
Aortic stenosis, valvular	22 (7.0%)	10 (6.1%)	11 (8.2%)
Aorto-left ventricular-Tunnel	1 (0.3%)	0 (0%)	1 (0.7%)
Atrial septal defect (ASD)	13 (4.4%)	10 (6.1%)	3 (2.2%)
Atrial septal defect—Sinus venosus type (SVASD)	1 (0.3%)	0 (0%)	1 (0.7%)
Atrioventricular septal defect (AVSD)	5 (1.7%)	4 (2.5%)	1 (0.7%)
Bicuspid Aortic Valve (BAV)	7 (2.4%)	3 (1.8%)	4 (3.0%)
Coarctation of the aorta (CoA)	32 (11%)	12 (7.4%)	20 (15%)
Common atrium	1 (0.3%)	1 (0.6%)	0 (0%)
Congenitally Corrected Transposition of the Great Arteries (ccTGA)	17 (5.7%)	8 (4.9%)	9 (6.7%)
Double aortic arch	1 (0.3%)	0 (0%)	1 (0.7%)
Double outlet right ventricle (DORV)	4 (1.3%)	3 (1.8%)	1 (0.7%)
Ebstein Anomaly (EA)	36 (12%)	27 (17%)	9 (6.7%)
Fabry-Disease	6 (2.0%)	4 (2.5%)	2 (1.5%)
Familial Thoracic Aortic Aneurysms and Dissections (TAAD)	1 (0.3%)	0 (0%)	1 (0.7%)
Hypoplastic Right Heart Syndrome (HRHS)	1 (0.3%)	1 (0.6%)	0 (0%)
Loeys-Dietz-Syndrome	2 (0.7%)	1 (0.6%)	1 (0.7%)
Marfan Syndrome	15 (5.1%)	9 (5.5%)	6 (4.5%)
Partial anomalous pulmonary venous return (PAPVR)	1 (0.3%)	0 (0%)	1 (0.7%)
Patent Ductus arteriosus Botalli (PDA)	1 (0.3%)	1 (0.6%)	0 (0%)
Pulmonary atresia with intact ventricular septal (PA-iVS)	1 (0.3%)	0 (0%)	1 (0.7%)
Pulmonary atresia with ventricular septal defect (PA-VSD)	9 (3.0%)	7 (4.3%)	2 (1.5%)
Pulmonary valve disease (PuV)	5 (1.6%)	4 (2.4%)	1 (0.7%)
Tetralogy of Fallot (ToF)	62 (21%)	31 (19%)	31 (23%)
Total anomalous pulmonary venous return (TAPVR)	3 (1.0%)	2 (1.2%)	1 (0.7%)
Transposition of the Great Arteries (TGA)	20 (6.7%)	10 (6.1%)	10 (7.5%)
Tricuspid Atresia (TA)	5 (1.7%)	3 (1.8%)	2 (1.5%)
Univentricular Heart (UVH)	10 (3.4%)	5 (3.0%)	5 (3.7%)
Ventricular septal defect (VSD)	10 (3.4%)	5 (3.1%)	5 (3.7%)

^1^ n (%).

**Table 3:** Overview of Anatomical, Functional, and Surgical Characteristics in Adults with Congenital Heart Disease, with Sex-Specific Distributions.

	Total (n = 297)	Female (n = 163)	Male (n = 134)	*p*-Value
Systemic ventricular morphology				0.7 ^1^
Right	37 (12%)	18 (11%)	19 (14%)	
Left	245 (82%)	137 (84%)	108 (81%)	
Univentricular	14 (4.7%)	7 (4.3%)	7 (5.2%)	
Undetermined	1 (0.3%)	1 (0.6%)	0 (0%)	
Cyanosis				
Acyanotic	183 (62%)	101 (62%)	82 (61%)	0.9 ^2,a^
Primary Cyanotic	114 (38%)	62 (38%)	52 (39%)	0.9 ^2,a^
Secondary Cyanotic	8 (2.7%)	7 (4.3%)	1 (0.7%)	0.077 ^1,a^
Treatment Status				0.055 ^1^
Palliative	9 (3.0%)	7 (4.3%)	2 (1.5%)	
Repair/Correction	219 (74%)	110 (67%)	109 (81%)	
Interventional	18 (6.1%)	12 (7.4%)	6 (4.5%)	
Native	51 (17%)	34 (21%)	17 (13%)	
Operation type				
Fallot repair	63 (21%)	31 (19%)	32 (24%)	0.38 ^2,a^
Fontan Typ	9 (3%)	3 (1.8%)	6 (4.5%)	0.30 ^1,a^
Valve replacement	68 (23%)	28 (17%)	40 (30%)	0.014 * ^,**2**,**a**^
Rastelli	3 (1%)	2 (1.2%)	1 (0.7%)	>0.9 ^1,a^
Ross	3 (1%)	2 (1.2%)	1 (0.7%)	>0.9 ^1,a^
Artial switch (Senning/Mustard)	21 (7.1%)	11 (6.7%)	10 (7.5%)	0.82 ^1,a^
Other	73 (25%)	42(26%)	31 (23%)	0.69 ^2,a^

Data is presented as n (%); * statistically significant (*p* < 0.05); ^1^ Fisher’s exact test; ^2^ Pearson’s Chi-squared test; ^a^ dichotomous variable.

**Table 4:** Overview of Cardiovascular and Non-Cardiac Comorbidities in Adults with Congenital Heart Disease and Heart Failure, Including Sex-Specific Distributions.

	Total (n = 297)	Female (n = 163)	Male (n = 134)	*p*-Value
**Cardiovascular Comorbidities**				
Aortopathy	164 (55%)	77 (47%)	87 (65%)	0.003 * ^,^ **^2^** ^,^ **^a^**
Arterial hypertension	111 (37%)	56 (34%)	55 (41%)	0.29 ^2,a^
Arrhythmias	99 (33%)	62 (38%)	37 (28%)	0.076 ^2,a^
Pulmonary hypertension	16 (5.4%)	13 (8.0%)	3 (2.2%)	0.055 ^2,a^
Cyanosis	12 (4.0%)	9 (5.5%)	3 (2.2%)	0.25 ^2,a^
Infective endocarditis	9 (3.0%)	5 (3.1%)	4 (3.0%)	1 ^1,a^
Coronary artery disease	7 (2.4%)	3 (1.8%)	4 (3.0%)	0.7 ^1,a^
**Non-cardiac Comorbidities**				
Thyroid dysfunction	73 (25%)	51 (31%)	22 (16%)	0.005 * ^,^ **^2^** ^,^ **^a^**
Hyperlipidemia/Lp(a) elevation	65 (22%)	36 (22.3%)	29 (21.2%)	
Hyperlipidemia	55 (19%)	29 (18%)	26 (19%)	0.84 ^2,a^
Lp(a) elevation	10 (3.4%)	7 (4.3%)	3 (2.2%)	0.52 ^1,a^
Renal failure	52 (18%)	33 (20%)	19 (14%)	0.22 ^2,a^
Neurological disorders	38 (13%)	13 (8.0%)	25 (19%)	0.010 * ^,^ **^2^** ^,^ **^a^**
Hyperuricemia	38 (13%)	18 (11%)	20 (15%)	0.41 ^2,a^
Anemia	31 (10%)	22 (13%)	9 (6.7%)	0.087 ^2,a^
Liver failure	31 (10%)	17 (10%)	14 (10%)	1 ^2,a^
Diabetes mellitus	22 (7.4%)	12 (7.4%)	10 (7.5%)	1 ^2,a^
Lung disease	19 (6.4%)	9 (5.5%)	10 (7.5%)	0.66 ^2,a^
Thromboembolic events	13 (4.4%)	8 (4.9%)	5 (3.7%)	0.84 ^2,a^
Sleep apnea syndrome	11 (3.7%)	5 (3.1%)	6 (4.5%)	0.55 ^1,a^
Syndromic disease	3 (1.0%)	3 (1.8%)	0 (0%)	0.25 ^1,a^

Data is presented as n (%); * statistically significant (*p* < 0.05); ^1^ Fisher’s exact test; ^2^ Pearson’s Chi-squared test; ^a^ dichotomous variable.

**Table 5:** Overview of Cardiovascular and Supportive Medication use in Adults with Congenital Heart Disease and Heart Failure, by Sex.

	Total (n = 297)	Female (n = 163)	Male (n = 134)	*p*-Value ^2^
No medication	31 (10%)	17 (10%)	14 (10%)	1 ^2,a^
Beta-blocker	185 (62%)	105 (64%)	80 (60%)	0.48 ^2,a^
Diuretics	103 (35%)	62 (38%)	41 (31%)	0.22 ^2,a^
Mineralocorticoid receptor antagonists (MRAs)	75 (25%)	41 (25%)	34 (25%)	1 ^2,a^
AT blocker	57 (19%)	27 (17%)	30 (22%)	0.26 ^2,a^
SGLT2 inhibitors	50 (17%)	29 (18%)	21 (16%)	0.74 ^2,a^
ACE inhibitor	47 (16%)	22 (13%)	25 (19%)	0.29 ^2,a^
Calcium channel blocker (CCB)	23 (7.7%)	12 (7.4%)	11 (8.2%)	0.96 ^2,a^
Angiotensin receptor-neprilysin inhibitor (ARNI)	25 (8.4%)	8 (4.9%)	17 (13%)	0.028 * ^,^ **^2^** ^,^ **^a^**
Digitalis glycosides	18 (6.1%)	8 (4.9%)	10 (7.5%)	0.50 ^2,a^
PDE5 inhibitors	11 (3.7%)	11 (6.7%)	0 (0%)	0.001 * ^,^ **^1^** ^,^ **^a^**
Endothelin receptor antagonists	15 (5.1%)	13 (8.0%)	2 (1.5%)	0.023 * ^,^ **^2^** ^,^ **^a^**
sGC stimulators	2 (0.7%)	2 (1.2%)	0 (0%)	0.50 ^1,a^
Anticoagulation	154 (52%)	75 (46%)	79 (59%)	0.035 * ^,^ **^2^** ^,^ **^a^**
Prostacyclin (IP) receptor agonists	1 (0.3%)	1 (0.6%)	0 (0%)	1 ^1,a^
Antiarrhythmic agents	19 (6.4%)	11 (6.7%)	8 (6.0%)	0.97 ^2,a^
Thyroid medication	91 (31%)	66 (40%)	25 (19%)	<0.001 * ^,^ **^2^** ^,^ **^a^**
Statins	59 (20%)	22 (13%)	37 (28%)	0.004 * ^,^ **^2^** ^,^ **^a^**
Iron	9 (3.0%)	7 (4.3%)	2 (1.5%)	0.19 ^1,a^

Data is presented as n (%); * statistically significant (*p* < 0.05); ^1^ Fisher’s exact test; ^2^ Pearson’s Chi-squared test; ^a^ dichotomous variable.

## Data Availability

The original contributions presented in this study are included in the article.

## Abbreviations

ACC/AHA	American College of Cardiology/American Heart Association
ACHD	Adults with Congenital Heart Disease
ACE inhibitor (ACEi)	Angiotensin-Converting Enzyme inhibitor
ARB/AT1 antagonist	Angiotensin II Type-1 Receptor blocker
ARNI	Angiotensin Receptor–Neprilysin Inhibitor
ASD	Atrial Septal Defect
AVSD	Atrioventricular Septal Defect
BAV	Bicuspid Aortic Valve
BMI	Body Mass Index
ccTGA	Congenitally Corrected Transposition of the Great Arteries
CCB	Calcium Channel Blocker
CHD	Congenital Heart Disease
CoA	Coarctation of the Aorta
DORV	Double Outlet Right Ventricle
EA	Ebstein’s Anomaly
FC	Functional Class (Perloff)
GPP	Good Pharmacoepidemiologic Practice
HF	Heart Failure
HRHS	Hypoplastic Right Heart Syndrome
IP (prostacyclin) receptor agonist	Prostacyclin (IP) receptor agonist
Lp(a)	Lipoprotein(a)
MRA	Mineralocorticoid Receptor Antagonist
PAPVR	Partial Anomalous Pulmonary Venous Return
PA-IVS	Pulmonary Atresia with Intact Ventricular Septum
PA-VSD	Pulmonary Atresia with Ventricular Septal Defect
PATHFINDER-CHD	(Registry name) PATHFINDER Registry in Congenital Heart Disease
PDE5 inhibitor	Phosphodiesterase-5 inhibitor
PDA	Patent Ductus Arteriosus (Botalli)
PuV	Pulmonary Valve (disease)
sGC stimulator	Soluble Guanylate Cyclase stimulator
SVASD	Sinus Venosus Atrial Septal Defect
TA	Tricuspid Atresia
TAAD	Thoracic Aortic Aneurysms and Dissections (familial)
TAPVR	Total Anomalous Pulmonary Venous Return
TGA	Transposition of the Great Arteries
ToF	Tetralogy of Fallot
UVH	Univentricular Heart
VSD	Ventricular Septal Defect
